# Supplementary material for: Assessment of the variant prioritization strategy for genomic newborn screening in the Generation Study
Source: Genet Med. 2025 Oct;27(10):101532. doi: 10.1016/j.gim.2025.101532 (PMC12714140; doi:10.1016/j.gim.2025.101532)
Supplement: Supplementary tables figures [file mmc1.pdf]

Supplemental Figures and Tables

Figures

Supplemental Figure 1: Coverage metrics per gene across the control-like subset aligned to DRAGEN v4 and across all exons.. Genes with metrics below the shown thresholds are labeled. For the figures b,d and f which show metrics for all exons, the corresponding gene is labelled as opposed to the exact exon for readability. The metrics are as follows: a) median coverage per gene (b) median coverage per exon (c) mean coverage per gene (d) mean coverage per exon (e) proportion of gene with coverage >15X (f) proportion of exon with coverage >15X .

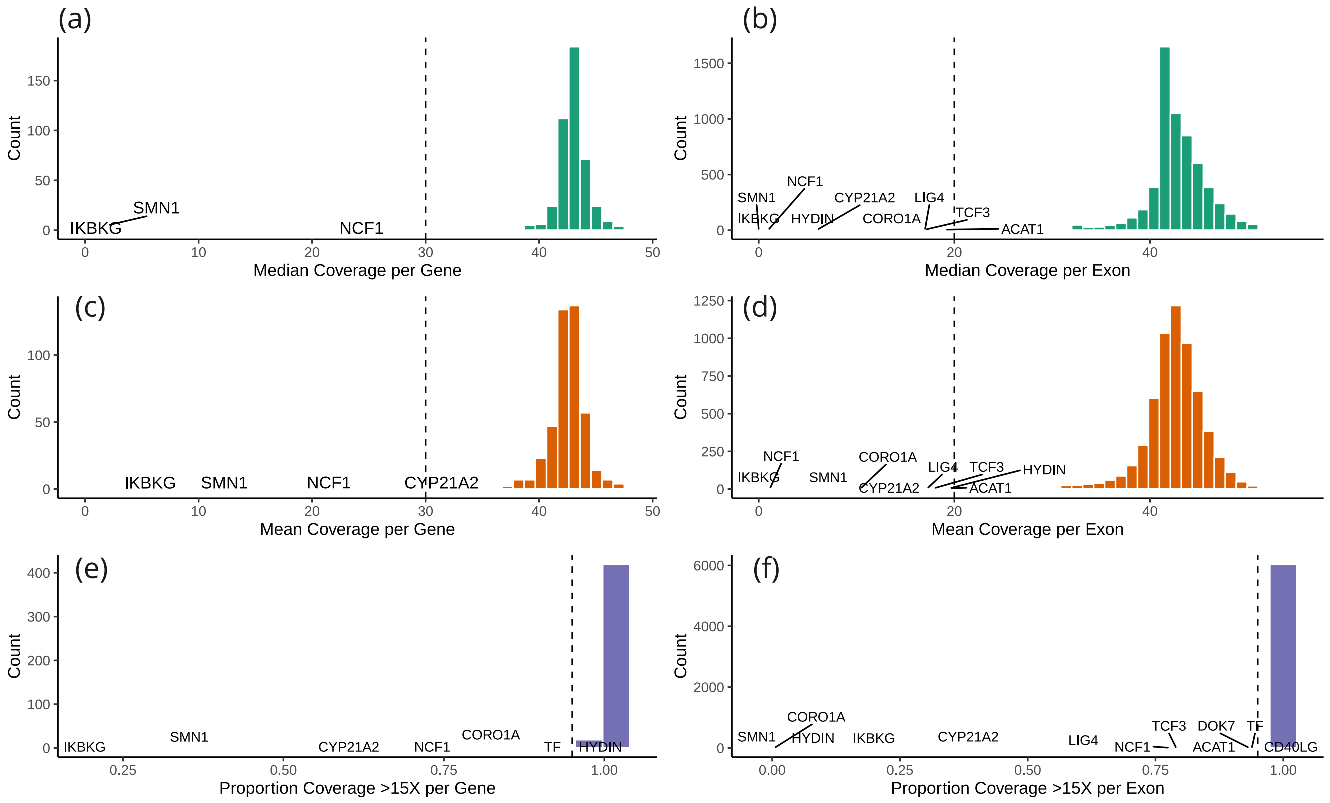

Supplemental Figure 2: Effect of different prioritisation rules for ClinVar variants with conflicting interpretations of pathogenicity (CIP). (a) Specificity of automated variant prioritisation in the control-like cohort (b) Sensitivity of automated variant prioritisation in the participants with diagnostic variants in genes included in the Generation Study from 100,000 Genomes Project and NGS Genome Medicine Service. Rules applied are detailed in the methods, thresholds on number of classifications. (P/LP = Pathogenic/Likely Pathogenic, B/LB = Benign Likely Benign)

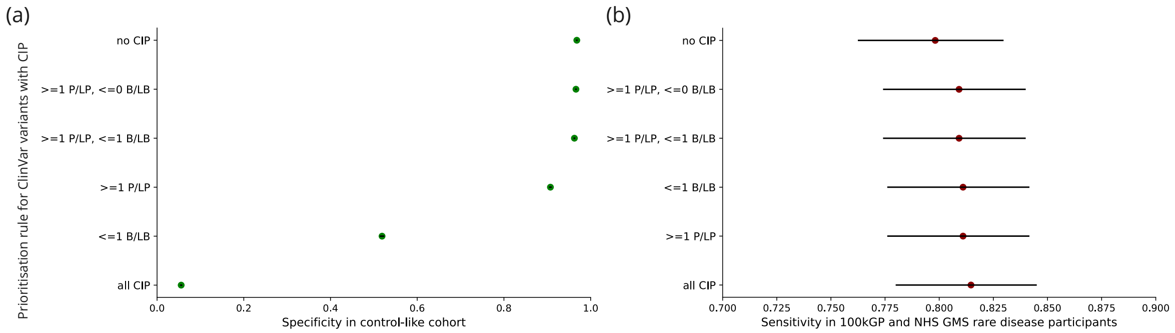

Supplemental Figure 3: Specificity of automated variant prioritisation. Comparing gene specificity in control-like cohort (DRAGEN v3.2) and (a) the control like subset realigned with DRAGEN v4.0 (b) replication cohort which is made up of participants recruited with cancer. The colour indicates whether the gene is also associated with cancer. (c) The number of samples with variants prioritised in the control-like cohort by prioritisation source.

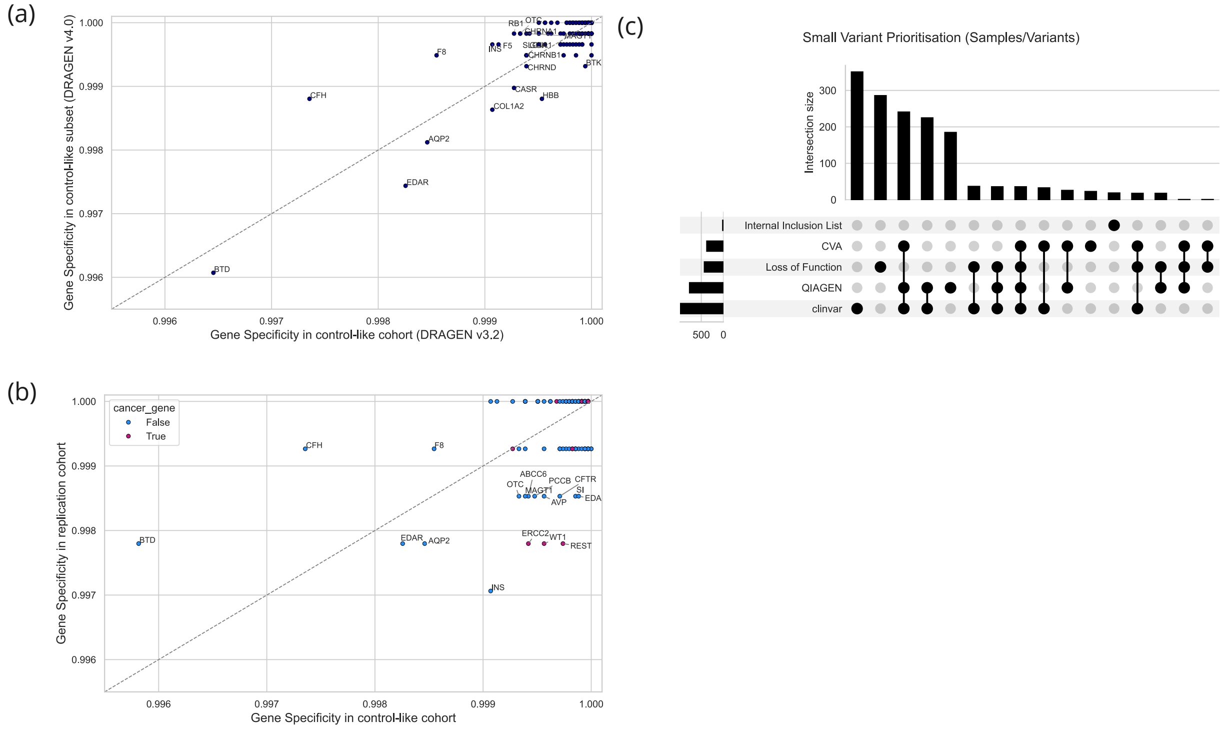

Supplemental Figure 4: Counts of prioritised diagnostic variants by prioritisation source in the 100,000 Genome Project and NHS GMS rare disease participants.

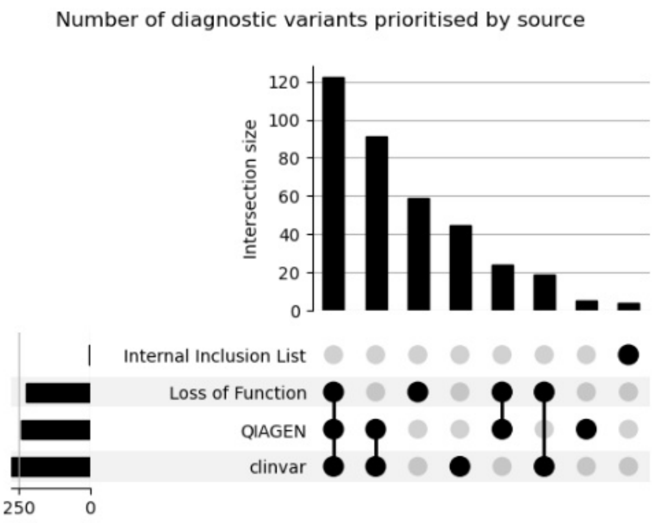

Tables

Supplemental Table 1: Principles for selecting conditions in the Generation Study

Principle	Description	Additional considerations
A	There is strong evidence that the genetic variant(s) causes the condition and can be reliably detected.	Where appropriate, there may be a confirmatory test that can establish whether or not the child has the condition.
B	A high proportion of individuals who have the genetic variant(s) would be expected to have symptoms that would have a debilitating impact on quality of life if left undiagnosed.	The impact on quality of life should consider factors such as the testimony of patients and families affected including social and environmental factors, and quality-adjusted life years (QALYs) where available.
C	Early or pre-symptomatic intervention for the condition has been shown to lead to substantially improved outcomes in children, compared to intervention after the onset of symptoms.	The intervention would normally be initiated in early childhood (by age 5); and could either cure, delay or modify the course of the condition.
D	Conditions screened for are only those for which the interventions are equitably accessible for all.	Incorporating input from NHS England and other relevant clinical and commissioning bodies.

Supplemental Table 2: Gene-condition pairs assessed for analytical validity (CSV file)
gene_name: HGNC gene name.
newborn_phenotype: Associated phenotype considered for inclusion in the Generation Study.
mode_of_inheritance: Mode of inheritance for the associated condition.
location_GrCH38: Genomic location of the gene (GRCh38).
ensembl_id : Ensembl gene ID.
included_in_GS: whether the gene-condition pair was included in the Generation Study.
internal_inclusion: whether there is an internal inclusion variant list only for this gene
lof_prioritisation: whether loss-of-function variant prioritisation was implemented in the pipeline.
targeted_caller: whether a targeted variant caller was assessed for this gene.

Supplemental Table 3: Ancestry composition of control-like cohort compared to the UK Office of National Statistics (ONS) live births occurring in 2020 by ethnicity (https://www.ons.gov.uk/). In the ONS statistics: European include White British and White Other, South Asian includes Bangladeshi, Indian and Pakistani, African includes Black African and Black Carribean and any other Black background. The ancestry projections were done by projecting principal components onto 1000 Genomes data (European (EUR), South Asian (SAS) and African (AFR)).

Ancestry	Control-like cohort inferred ancestry	ONS 2020 Live births (reported)
European	78.2%	70.6%
South Asian	7.8%	9.9%
African	4.3%	5.1 %

Supplemental Table 4: Coverage and CNV callability metrics for all genes considered (tsv file). Below is a description of each column:
hgnc_symbol: HGNC-approved gene symbol.
ensembl_gene_id: Unique Ensembl Gene ID for the gene.
ensembl_transcript_id: Unique Ensembl Transcript ID specifying the assessed transcript.
ensembl_transcript_id_version: Unique Ensembl Transcript ID version specifying the version of the assessed transcript.
location: Genomic coordinates (chromosome and start/end positions) of the gene or transcript.
total_bases: Total number of bases assessed within the transcript.
total_exons: Total number of exons in the transcript.
bases_less_10: Number of bases in the transcript with coverage below 10X.
percentage_less_10: Percentage of total bases in the transcript with coverage below 10X.
num_exons_less_10: Number of exons in the transcript where median coverage falls below 10X.
bases_less_15: Number of bases in the transcript with coverage below 15X.
percentage_less_15: Percentage of total bases in the transcript with coverage below 15X.
num_exons_less_15: Number of exons in the transcript where median coverage is below 15X.
bases_less_20: Number of bases in the transcript with coverage below 20X.
percentage_less_20: Percentage of total bases in the transcript with coverage below 20X.
num_exons_less_20: Number of exons in the transcript where median coverage is below 20X.
bases_less_25: Number of bases in the transcript with coverage below 25X.
percentage_less_25: Percentage of total bases in the transcript with coverage below 25X.
num_exons_less_25: Number of exons in the transcript where median coverage is below 25X

Supplemental Table 5: Internal Variant Inclusion List (csv file)
Columns: Chromosome, Position, Reference, Alternate, Published variant nomenclature, HGVS, Evidence (PMID to support inclusion of variant), Gene (HGNC)

Supplemental Table 6: Gene-condition pairs with internal inclusion lists included in the Generation Study. (xlsx file)

Supplemental Table 7: Variant exclusion list (xlsx file)
Columns: Gene (HGNC), Chromosome, Position, Reference, Alternate, HGVS

Supplemental Table 8: Allele frequency thresholds for gnomAD populations in pLoF prioritisation (gnomAD Genomes v3.1.2, gnomAD Exomes v2.1.1). These differ for gene-conditions associated with dominant vs recessive phenotypes.

Dataset	Population	Dataset size (individuals)	Monoallelic (dominant)	Biallelic (recessive)
Internal	Mixed	5,855	0.001	0.01
GNOMAD_GENOMES	African/African American	20,744	0.0005	0.01
GNOMAD_GENOMES	Latino/Admixed American	7,647	0.001	0.01
GNOMAD_GENOMES	Ashkenazi Jewish	1,736	0.003	0.01
GNOMAD_GENOMES	East Asian	2,604	0.002	0.01
GNOMAD_GENOMES	European (Finnish)	5,316	0.001	0.01
GNOMAD_GENOMES	Middle Eastern	158	0.1	0.1
GNOMAD_GENOMES	European (non-Finnish)	34,029	0.0005	0.01
GNOMAD_GENOMES	South Asian	2,419	0.002	0.01
GNOMAD_EXOMES	Latino/Admixed American	17,296	0.0005	0.01
GNOMAD_EXOMES	Ashkenazi Jewish	5,040	0.001	0.01
GNOMAD_EXOMES	East Asian	9,197	0.001	0.01
GNOMAD_EXOMES	European (Finnish)	10,824	0.001	0.01
GNOMAD_EXOMES	European (non-Finnish)	56,885	0.0005	0.01
GNOMAD_EXOMES	South Asian	15,308	0.001	0.01

Supplemental Table 9: Gene specificity across genes included in the Generation Study in the control-like cohort ('gene_specificity_controllike'), control-like subset ('gene_specificity_controllike_subset') and the replication cohort ('gene_specificity_replication'). Note that this only contains genes where a variant is prioritised, all genes not included in this table have specificity of 1 across all cohorts.

Supplemental Table 10: Genes and conditions included under different modes of inheritance and impact on gene specificity. Table describes genes where the modes of inheritance included in the Generation Study were changed due to issues in gene specificity.

Gene	Conditions included	Modes of Inheritance included	Number of prioritised variants	Number of prioritised samples	Gene specificity (%)
CHRNE	Myasthenic syndrome, congenital, 4, autosomal recessive; Myasthenic syndrome, congenital, 4, autosomal dominant	monoallelic and biallelic	25	219	99.37
	Myasthenic syndrome, congenital, 4, autosomal recessive	biallelic	2	1	99.99
ALPL	Autosomal recessive hypophosphatasia; Autosomal dominant hypophosphatasia	monoallelic and biallelic	55	183	99.47
	Autosomal recessive hypophosphatasia	biallelic	5	3	99.99
ABCC8	Generalized arterial calcification of infancy 2; Diabetes mellitus, permanent neonatal 3, autosomal recessive; Hyperinsulinemic hypoglycemia, familial, 1; Diabetes mellitus, permanent neonatal 3, autosomal dominant; ABCC9 associated hypertrichotic osteochondrodysplasia	monoallelic and biallelic	71	172	99.50
	Generalized arterial calcification of infancy 2 ; Diabetes mellitus, permanent neonatal 3, autosomal recessive; Hyperinsulinemic hypoglycemia, familial, 1	biallelic	0	0	100

Supplemental Table 11: Variants prioritised in the largest number of samples in the control-like cohort (xlsx file)

Supplemental Table 12: Potential compound heterozygous variants prioritised in the control-like cohort broken down by co-occurrence prediction in gnomAD. This is also subset to those that are >150bp apart.

Co-occurrence prediction	All potential compound heterozygous variants		Potential compound heterozygous variants >150bp apart	
	Number of samples	Number of unique variant pairs	Number of samples	Number of unique variant pairs
Different haplotye	64	45	50	40
Same haplotype	167	36	109	26
Uncertain	11	7	6	6
No prediction	100	69	49	39

Supplemental Table 13: Conditions associated with variants deemed reportable in control-like subset (xlsx file)

Supplemental Table 14: Sensitivity by mode of inheritance for 100kGP and NHS GMS rare disease participants with diagnostic variants in genes included in the Generation Study. Sensitivity also split by genes with internal inclusion lists.

Mode of Inheritance for condition included	Internal Inclusion list only genes	Total number of diagnostic variants	Number of prioritised diagnostic variants	Total samples	Number of Samples with >=1 variant prioritised	Sensitivity (%)
biallelic	False	411	326	279	230	82.44
	True	22	2	12	2	16.67
monoallelic	False	194	169	194	169	87.11
	True	13	2	13	2	15.38
X-linked biallelic	False	41	33	41	33	80.49
X-linked monoallelic	False	6	5	6	5	83.33

Supplemental Table 15: Predicted consequences of diagnostic variants that were not prioritised in 100kGP and NHS GMS rare disease participants with diagnostic variants in genes included in the Generation Study

Predicted Consequence of diagnostic variant	Number of variants not prioritised
Missense	54
Splice region	8
Inframe deletion	2
